# The Tissue Microlocalisation and Cellular Expression of CD163, VEGF, HLA-DR, iNOS, and MRP 8/14 Is Correlated to Clinical Outcome in NSCLC

**DOI:** 10.1371/journal.pone.0021874

**Published:** 2011-07-22

**Authors:** Chandra M. Ohri, Aarti Shikotra, Ruth H. Green, David A. Waller, Peter Bradding

**Affiliations:** 1 Department of Infection, Immunity and Inflammation, University of Leicester, Leicester, United Kingdom; 2 Department of Respiratory Medicine, Institute for Lung Health, Glenfield Hospital, Leicester, Leicester, United Kingdom; 3 Department of Thoracic Surgery, Glenfield Hospital, Leicester, Leicester, United Kingdom; University of Chicago, United States of America

## Abstract

**Background:**

We have previously investigated the microlocalisation of M1 and M2 macrophages in NSCLC. This study investigated the non-macrophage (NM) expression of proteins associated with M1 and M2 macrophages in NSCLC.

**Methods:**

Using immunohistochemistry, CD68^+^ macrophages and proteins associated with either a cytotoxic M1 phenotype (HLA-DR, iNOS, and MRP 8/14), or a non-cytotoxic M2 phenotype (CD163 and VEGF) were identified. NM expression of the markers was analysed in the islets and stroma of surgically resected tumours from 20 patients with extended survival (ES) (median 92.7 months) and 20 patients with poor survival (PS) (median 7.7 months).

**Results:**

The NM expression of NM-HLA-DR (p<0.001), NM-iNOS (p = 0.02) and NM-MRP 8/14 (p = 0.02) was increased in ES compared to PS patients in the tumour islets. The tumour islet expression of NM-VEGF, was decreased in ES compared to PS patients (p<0.001). There was more NM-CD163 expression (p = 0.04) but less NM-iNOS (p = 0.002) and MRP 8/14 (p = 0.01) expression in the stroma of ES patients compared with PS patients. The 5-year survival for patients with above and below median NM expression of the markers in the islets was 74.9% versus 4.7% (NM-HLA-DR p<0.001), 65.0% versus 14.6% (NM-iNOS p = 0.003), and 54.3% versus 22.2% (NM-MRP 8/14 p = 0.04), as opposed to 34.1% versus 44.4% (NM-CD163 p = 0.41) and 19.4% versus 59.0% (NM-VEGF p = 0.001).

**Conclusions:**

Cell proteins associated with M1 and M2 macrophages are also expressed by other cell types in the tumour islets and stroma of patients with NSCLC. Their tissue and cellular microlocalisation is associated with important differences in clinical outcome.

## Introduction

Lung cancer is responsible for more deaths worldwide than any other cancer [1] and non-small cell lung cancer (NSCLC) accounts for the majority of these cases. Even with optimal presentation characterised by good performance status and early tumour stage, the 5 year survival is only 67% [2]. Virchow was the first to note the presence of leucocytes in neoplastic tissues in 1863 and suggested a connection between inflammation and cancer [3]. It is now accepted that both innate and adaptive immunity play a major role in cancer development and modulation [4]–[6], and that most tumours arise within an immune-permissive environment [6]. However, the immune mechanisms regulating tumour development and progression are poorly understood at both the cell and molecular levels. Understanding and then manipulating these immunological pathways may offer novel approaches to cancer therapy [7].

One recurring weakness in many studies which have investigated the immune response within tumour tissues in the lung and elsewhere has been the tendency to analyse the tumour stroma and epithelial cell clusters (or islets) as a whole. However, the tissue microenvironment plays a major role in determining the phenotype and function of numerous structural and inflammatory cells. Thus the role of cells within the stroma is likely to be quite different to those in the tumour islets. In support of this statement, we have demonstrated previously that the microlocalisation of immune cells in tumour sub-compartments (islets or stroma) in NSCLC has a profound influence on survival [8]. Specifically, we noted a marked survival advantage for patients with increased numbers of macrophages in their tumour islets irrespective of tumour stage [8], a finding which has been confirmed by others in an independent cohort [9]. It is also well recognised that there are two major macrophage phenotypes [10]–[13], M1 (classically activated) and M2 (alternatively activated). We have shown previously in NSCLC that HLA-DR, inducible nitric oxide synthase (iNOS), myeloid related protein 8/14 (MRP 8/14) and tumour necrosis factor-alpha (TNFα) are markers of M1 (cytotoxic) macrophages and that CD163 and vascular endothelial growth factor (VEGF) are markers of M2 (non-cytotoxic) macrophages [14]. Of significance, we demonstrated that macrophages within NSCLC tumour islets are predominantly of the cytotoxic M1 phenotype and are associated with extended survival supporting the view that the survival advantage conferred by tumour islet macrophage infiltration is related to their cytotoxic potential.

HLA-DR [15], iNOS [16], MRP 8/14 [17] and TNFβ [18] have been associated with improved survival in NSCLC in previous studies. It was suggested that TNFα expression is not associated with survival [18], but we have shown recently that its expression within the tumour islets correlates with improved survival, while its expression in the tumour stroma predicts poor survival [19]. Because expression of these cell proteins by macrophages is linked strongly to NSCLC survival, we have now determined whether this applies to these markers expressed by other cell types.

## Methods

### Study Population

The study was approved by the Leicestershire Research Ethics Committee (approval reference number 6529). All patients gave their written consent at time of surgery for donation of tissue but informed consent was not obtained for each patient for this study as they had died at the time of analysis. The Leicestershire Research Ethics Committee approved the consent procedure for those who died. The tissue specimens evaluated were from 40 patients with NSCLC who had undergone resection with curative intent at the University Hospitals of Leicester National Health Service Trust (Leicester, United Kingdom). These patients had resections during two periods – one dating from 1991 to 1994 and the second from January to December 1999. This cohort of patients has been described previously [8] and these 40 patient samples examined before [14]. Patients were selected for the study based on their survival, without knowledge of their previous tumour cell counts. 20 patients had extended survival (ES) (mean 92.7 months, S.E.M.7.2), and 20 patients had poor survival (PS) (mean 7.7 months, S.E.M. 0.7). This method of patient selection has been used previously [14], [19], [20], [21], [22]. Patient characteristics are shown in Table 1.

**Table 1:** Patient characteristics.

Characteristic	Extended Survival	Poor Survival
No. of patients	20	20
Age – years	69.1±1.8	69.6±1.6
Male sex – no. (%)	16 (80)	11 (55)
Year of surgery – no. (%)		
1991	0 (0)	1 (5)
1992	3 (15)	1 (5)
1993	2 (10)	1 (5)
1994	1 (5)	1 (5)
1999	14 (70)	16 (80)
Tumour stage – no. (%)		
1	13 (65)	13 (65)
2	5 (25)	3 (15)
3a	2 (10)	3 (15)
3b and 4	0 (0)	1 (5)
Histology – no. (%)		
Squamous	14 (70)	10 (50)
Adenocarcinoma	4 (20)	5 (25)
Large cell	0 (0)	3 (15)
Other	2 (10)	2 (10)
Tumour Grade – no. (%)		
Well	2 (10)	0 (0)
Moderate	8 (40)	1 (5)
Poor	11 (55)	18 (90)
Not recorded	1 (5)	1 (5)
Adjuvant Chemotherapy (%)	0 (0)	0 (0)
Radiotherapy (%)	3 (15)	1 (5)
Palliative Radiotherapy (%)	2 (10)	1 (5)
Survival – months	92.7±7.2	7.7±0.7
Complete resection*	16	14

*Plus-minus values are means ± SEM.*

*information on resection margins available from 18 Extended Survival patients and 17 Poor Survival patients.

### Immunohistology

The specimens studied were formalin fixed and paraffin embedded. Only blocks containing the advancing edge of the tumour were evaluated. Tissue sections of 4 µm thickness were cut onto glass slides and then de-waxed in xylene and rehydrated through graded alcohols. Antigen retrieval was carried out using Trilogy Antigen Retrieval solution (Cell Marque, Hot Springs, United States of America) in a pressure cooker (heated to 117.5°C for 1 min and then cooled to 100°C for 30 seconds). Mouse antihuman macrophage CD68 mAb (clone PGM1; Dakocytomation, Ely, Cambridgeshire, United Kingdom) was used as a specific marker for macrophages. Antibodies for phenotype were all mouse antihuman as follows: i) CD163 mAb (clone 10D6, Novocastra, United Kingdom), ii) HLA-DR mAb (clone TAL.1B5; Hycult biotechnology, the Netherlands), iii) iNOS mAb (clone 2D2-B2; R&D systems, Abingdon, United Kingdom), iv) MRP 8/14 mAb (clone 27E10; Bachem Distribution Services, Germany), and vi) VEGF mAb (clone 14–124; Abcam, Cambridge, United Kingdom). IgG1 mAb (clone DAK-GO1; Dakocytomation) was used as an isotype control. Immunostaining for CD68 and each individual phenotype marker was performed using the Envision double-stain kit (Dakocytomation) according to the manufacturer's instructions and as described previously(8). Thus, six slides were prepared for each patient – CD68 versus CD163, CD68 versus HLA-DR, CD68 versus iNOS, CD68 versus MRP 8/14, and CD68 versus VEGF. CD68 was developed with peroxidase and 3,3′-diaminobenzidine tetrahydrochloride (brown reaction product), and each phenotype marker with alkaline phosphatase and fast red (red reaction product). Sections were then counterstained with haematoxylin and mounted in an aqueous mounting medium (BDH Chemicals Ltd, Poole, United Kingdom). Appropriate isotype controls were performed where the primary antibodies were replaced by irrelevant mouse mAb of the same isotype and at the same concentration as the specific primary mAb.

### Analysis and Validation of Immunostaining

Analysis was performed blind with respect to the clinical outcome. The ten most representative high-power fields (×400) per slide were manually selected using an Olympus BX50 microscope (Olympus, Southall, United Kingdom). The respective areas of stroma and of tumour-cell islets were then measured at ×400 magnification using Scion image analysis software (Based on National Institutes of Health Image for Macintosh, modified for Windows [Scion Corp, Frederick, MD]). The number of nucleated macrophages and cells with positive staining for the phenotype marker in each area were then counted manually and expressed as cells/mm^2^ of stroma or tumour islets. Analysis was validated previously [8], [14], [19], [20].

### Statistical Analysis

Statistical analyses were carried out using the GraphPad Prism software package (v. 4.02; GraphPad Prism Software Inc, San Diego, CA). For categoric analysis, the median value was used as a cut point to dichotomise the series. The χ^2^ test was used to test for relationships between categoric variables, and the Mann-Whitney nonparametric test was used to compare categoric with continuous variables. The Kruskal-Wallis one-way analysis of variance test was used to compare multiple groups. Kaplan-Meier survival curves were used to look for correlations with survival and were compared with the use of the log-rank statistic. For the above comparisons, p<0.05 was considered statistically significant.

## Results

### Patient Characteristics

These are shown in table 1. Of note, none of the patients received adjuvant chemotherapy as this was the approach at time of surgical resections in this study.

### Validation of Analysis

**Figure 1:**
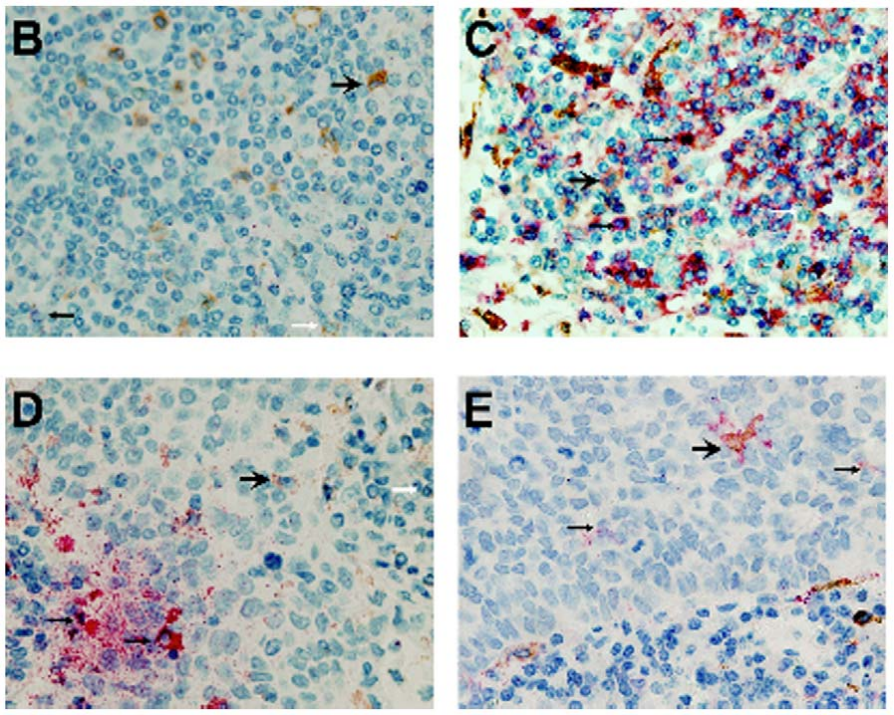
Immunohistology in NSCLC. In (A)–(E), CD68^+^ cells are stained brown and (A) CD163 (red), (B) VEGF (red), (C) HLA-DR (red), (D) iNOS (red) and (E) MRP 8/14 (red). Singlestain CD68 brown (white arrowhead), Singlestain non-macrophage phenotypic marker red (small arrowhead), Double-stain cells contain both brown and red (large arrowhead).

Clear and distinguishable staining was evident for both CD68 and each phenotype marker. In addition to the double-stained macrophages described previously [14], numerous single-stained cells which were not macrophages were readily identifiable (Fig. 1A–E). Appropriate isotype controls were negative. Cell counts were repeated and an intraclass correlation coefficient was calculated as 0.998 (p<0.001). This method of analysis has also been validated by our group previously [8].

### Cellular Distribution in Tumour Islets and Stroma

**Figure 2:**
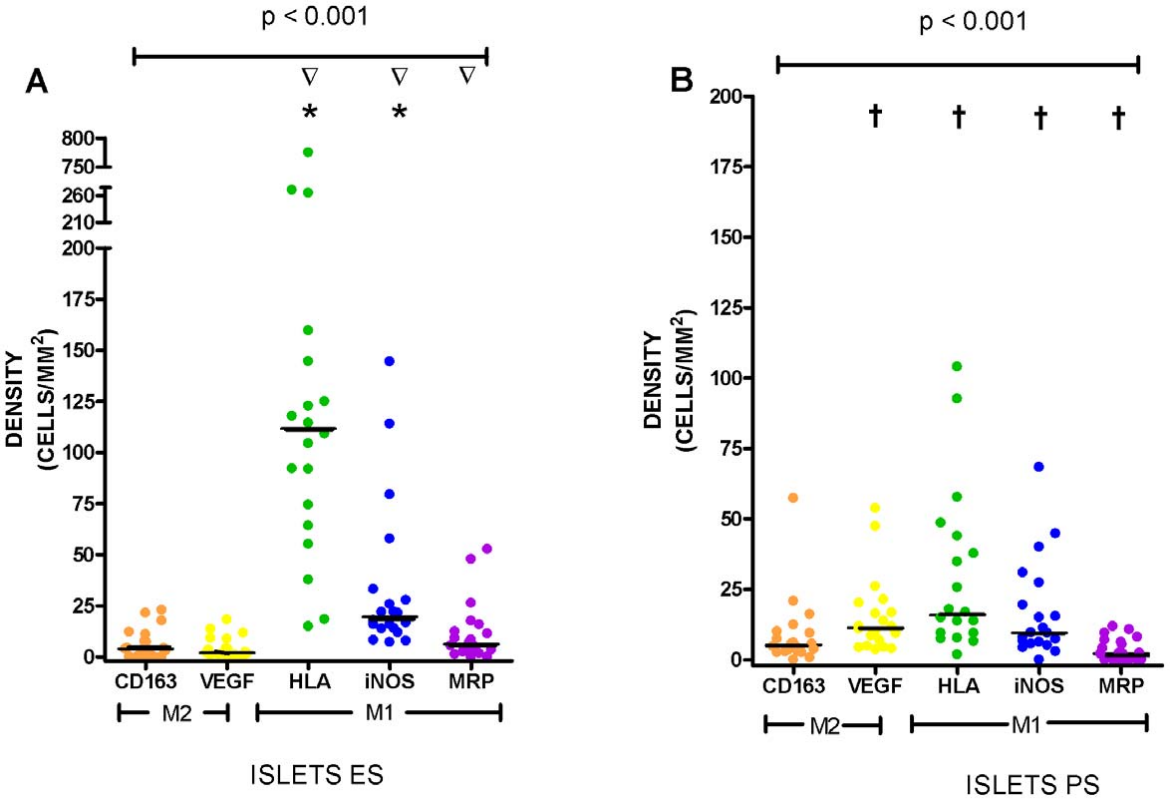
Non-macrophage double-stain densities in the islets in (A) extended survival (ES) and (B) poor survival (PS). * represents a significant difference (p<0.05) in expression compared with CD163. **∇** represents a significant difference (p<0.05) in expression compared with VEGF. **†** represents a significant difference (p<0.05) between the corresponding marker in the ES group.

Non-macrophage (NM) expression of CD163 (NM-CD163) in the tumour islets was similar when compared between ES and PS patients (median 3.9 versus 5.2 cells/mm^2^ respectively, p = 0.39), but expression of NM-VEGF was significantly reduced in the ES compared to PS patients (2.1 versus 11.4 cells/mm^2^ respectively, p<0.001). In contrast, when comparing the islets of ES versus PS patients, expression of NM-HLA-DR (111.7 versus 15.9 cells/mm^2^ respectively, p<0.001), NM-iNOS (19.7 versus 9.6 cells/mm^2^ respectively, p = 0.02), and NM-MRP 8/14 (6.3 versus 2.2 cells/mm^2^ respectively, p = 0.02) was significantly elevated in the ES islets. There was significantly less expression of NM-CD163 in the islets of ES patients compared with expression of NM-HLA-DR and NM-iNOS (p<0.001, and p<0.001respectively) (Fig. 2A). There was also significantly less expression of NM-VEGF in the islets of ES patients compared to NM-HLA-DR, NM-iNOS, and NM-MRP 8/14 (p<0.001, p<0.001, and p = 0.04 and, respectively).

**Figure 3:**
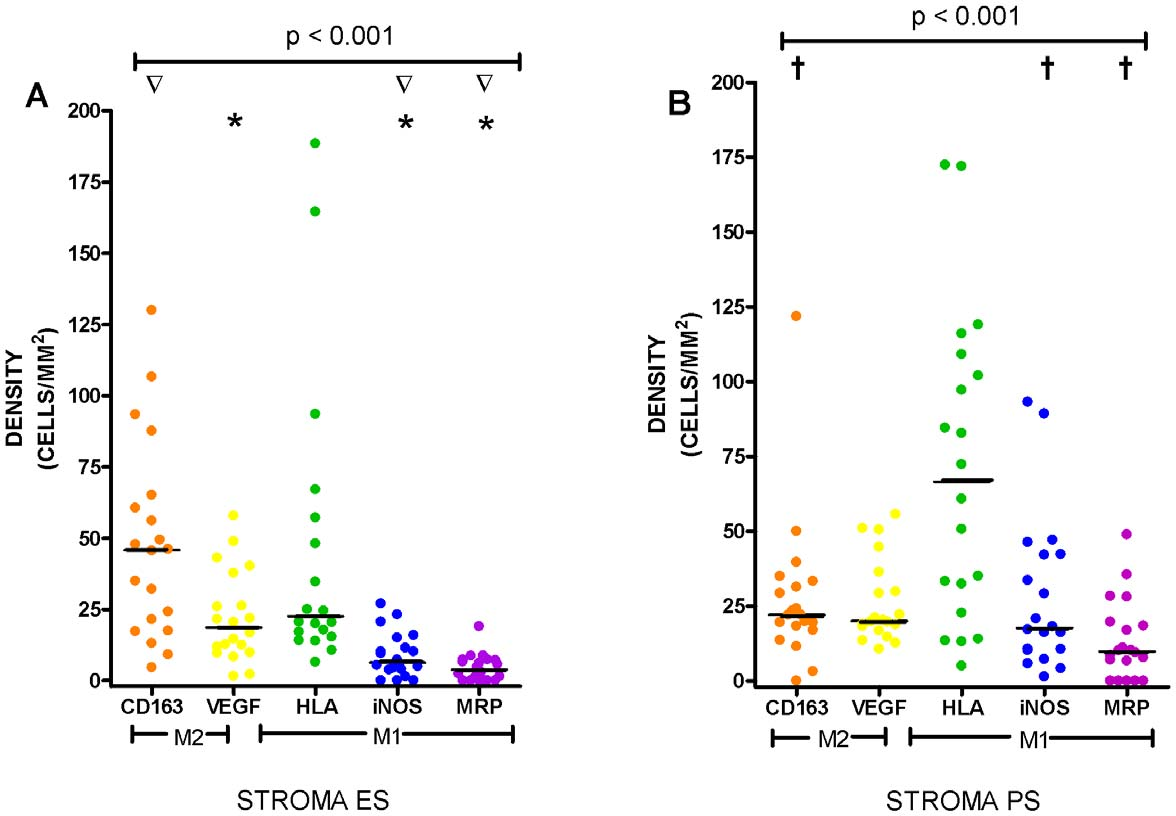
Non-macrophage double-stain densities in the stroma in (A) extended survival (ES), and (B) poor survival (PS). * represents a significant difference (p<0.05) in expression compared with CD163. **∇** represents a significant difference (p<0.05) in expression compared with VEGF. **†** represents a significant difference (p<0.05) between the corresponding marker in the ES group.

With respect to the tumour stroma, there was higher expression in the stroma of ES patients compared with PS patients for NM-CD163 (45.9 versus 22.2 cells/mm^2^) (p = 0.04) and lower expression in the stroma of ES compared to PS patients for NM-iNOS (6.4 versus 17.7 cells/mm^2^, p = 0.002) and NM-MRP 8/14 (3.9 versus 9.8 cells/mm^2^, p = 0.01) (Figs. 3A and B). There was significantly higher expression of NM-CD163 in the stroma of ES patients compared with expression of NM-iNOS and NM-MRP 8/14 (p<0.001) (Fig. 3A). There was also significantly higher expression of NM-VEGF in the stroma of ES patients compared to NM-iNOS and NM-MRP 8/(p = 0.003 and p<0.001, respectively).

In the ES group there was higher expression of NM-CD163 and NM-VEGF (p<0.001) in the stroma compared to the islets and conversely higher expression of HLA-DR and iNOS in the islets compared to the stroma (p = 0.002 and p<0.001) (Figs. 2A and 3A). The results are summarised in Table 2.

**Table 2:** Summary of the cellular distribution results in the tumour islets and stroma indicating the median density of cells/mm^2^ in extended survival (ES) and poor survival (PS) patients.

	Islets ES	Islets PS	Stroma ES	Stroma PS
**CD163**	3.9	5.2	45.9‡	22.2
**VEGF**	2.1*	11.4	18.6	20.1
**HLA-DR**	111.7*	15.9	22.6	66.6
**iNOS**	19.7*	9.6	6.4‡	17.7
**MRP 8/14**	6.3*	2.2	3.9‡	9.8

*p<0.05 compared to islets PS.

‡ p<0.05 compared to stroma PS.

### Kaplan-Meier Survival Analysis

**Figure 4:**
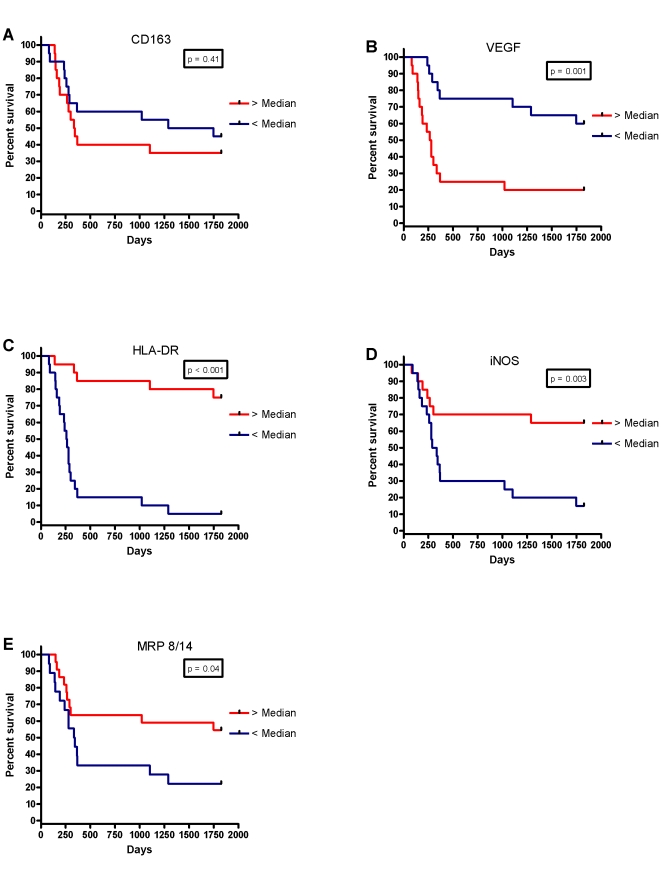
Kaplan-Meier five year survival curves for non-macrophage densities in the tumour islets for (A) CD163, (B) VEGF, (C) HLA-DR, (D) iNOS and (E) MRP 8/14.

**Figure 5:**
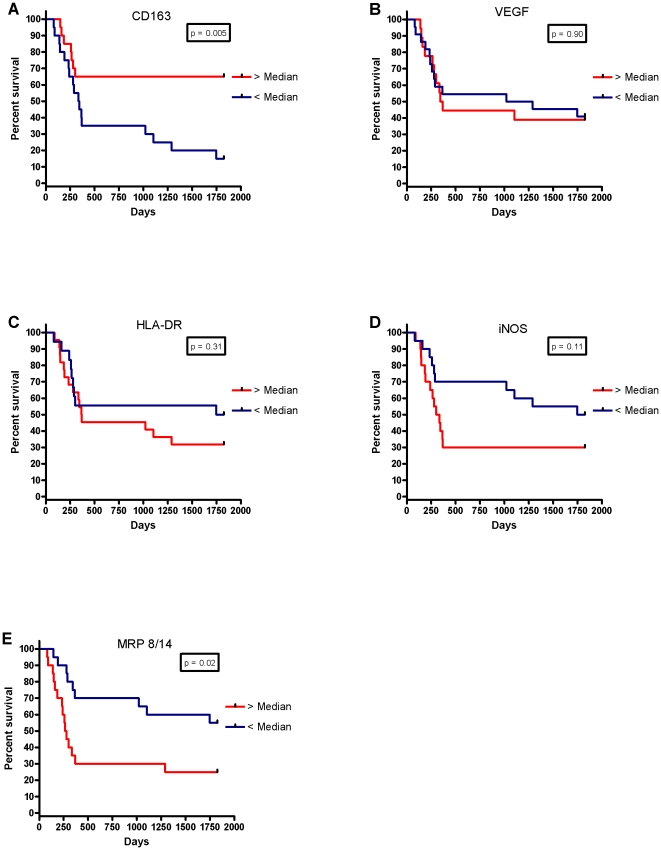
Kaplan-Meier five year survival curves for non-macrophage densities in the tumour stroma for (A) CD163, (B) VEGF, (C) HLA-DR, (D) iNOS and (E) MRP 8/14.

For further analysis, the data were divided into two groups above and below the median cell-count values and Kaplan-Meier survival curves were plotted. Looking at NM staining for each protein, there was an inverse relationship between tumour islet density and survival for NM-VEGF (p<0.001), and significant positive associations for NM-HLA-DR (p<0.001), NM-iNOS (p = 0.003) and NM-MRP 8/14 (p = 0.04) (Fig. 4). There was also an association between survival and tumour stroma NM-CD163 density (p = 0.005), but an inverse relationship for MRP 8/14 (p = 0.02) (Fig. 5).

## Discussion

Macrophage infiltration of tumour islets in surgically resected NSCLC confers a marked survival advantage independently of tumour stage, while increasing numbers of macrophages in the tumour stroma are associated with a worse prognosis [8]. We have also shown previously that there are two distinct macrophage phenotypes in NSCLC tissue: M1 macrophages that express HLA-DR, iNOS, MRP 8/14 and TNFα, and M2 macrophages that express CD163 and VEGF [14]. We also noted that macrophages within NSCLC tumour islets are predominantly of the cytotoxic M1 phenotype and are associated with extended survival. The protein markers for macrophages are also known to be expressed by other cell types, and the biological activity of these proteins may therefore extend beyond their role in macrophage biology. We have therefore examined the non-macrophage (NM) expression of markers associated with M1 and M2 macrophages in surgically resected NSCLC specimens. Our patients were divided into two groups according to survival. The clinical characteristics of the patients in both groups were well balanced with the exception of tumour grade which was slightly worse in the poor survival group.

It was clear that cells other than macrophages expressed CD163, VEGF, HLA-DR, iNOS, and MRP 8/14 in both the NSCLC tumour islets and stroma. The results were very interesting in that the expression of NM-VEGF was lower in the tumour islets of the ES compared to PS patients, but expression of NM-HLA-DR, NM-iNOS and NM-MRP 8/14 was increased in the tumour islets of ES versus PS patients. This suggests that these proteins play an important role in tumour immunology and disease progression.

The increased expression in the tumour islets of NM-HLA-DR, NM-iNOS and NM-MRP 8/14 in patients with ES compared to PS, mirrors the findings with the macrophage expression of these markers [14]. These proteins were also relatively increased in the ES islets compared to ES stroma, a feature reversed in the PS patients. This suggests that NM-HLA-DR, NM-iNOS and NM-MRP 8/14 must be expressed in the islets to fulfil their anti-tumourigenic potential.

In contrast, macrophages expressing VEGF are increased in the islets of ES patients, but NM-VEGF is significantly increased in the islets of PS patients. Thus the biological outcome with respect to VEGF expression is critically related to the cells expressing it and their location within the tumour. We have observed similar findings with respect to TNFα expression [19]. The important clinical message from these observations is that if VEGF for example is to be neutralised with novel biological therapies, it would seem prudent to target the population who are islet macrophage-VEGF^low^, islet NM-VEGF^high^.

The major limitation of this study that will need addressing in future work is that we have not identified the cells expressing CD163, VEGF, HLA-DR, iNOS and MRP 8/14 as there are numerous permutations. In addition the biological role of many of these proteins are poorly understood, and it is uncertain whether their association with survival is due their own function, or whether they are markers of other pro/anti-tumourigenic activities performed by the cells expressing them. CD163 is expressed by differing organ-specific macrophages, including alveolar and interstitial macrophages in the lung [23], and dendritic cells [24], and is a member of the scavenger receptor family which removes haemoglobin complexes from the tissue microenvironment [25]. It may play a role in chronic inflammation [26] but its role in cancer remains unclear. Nevertheless, its expression by macrophages in the NSCLC tumour islets is associated with improved outcome following surgery, and as shown here, its NM-expression in the stroma also associates with ES.

VEGF is a pro-angiogenic molecule secreted in various isoforms which acts by binding to tyrosine kinase receptors. VEGF has been implicated in the process of tumour angiogenesis leading to tumour proliferation [27], [28], but also exerts important effects on cellular function such as proliferation and migration [27] and so it is easy to envisage why increased NM-VEGF in the tumour islets would be associated with poor survival.

HLA-DR is expressed by diverse cells including mast cells [29], natural killer cells [30] and epithelial cells. Its main function is in antigen presentation to elicit an immune response. Monocytes activated by tumour-derived microvesicles from pancreatic, colon and lung cancer cell lines have been found to show increased expression of HLA-DR and a resulting increase in production of reactive oxygen intermediates and TNFα [31]. It is known that HLA-DR is expressed by natural killer cells and it is therefore possible that the survival advantage conferred by high non-macrophage expression of HLA-DR relates to the presence of natural killer cells in the tumour islets. Again this is compatible with our previous findings suggesting that when cytotoxic cells, for example macrophages [14], are present in the tumour islets, patients have improved survival.

MRP 8/14 expression by macrophages is associated with the release of TNFα [32]. MRP 8/14 is also known to be expressed by neutrophils [33]. The antigen for MRP 8/14 has been found only in inflammatory tissues and shown to be absent from normal resident mononuclear phagocytes [34]. MRP 8/14 is enhanced by IFN-γ and LPS [34], [35] and has bacteriostatic properties [36]. iNOS has a role in conveying protection against bacteria, viruses and parasites as well as suppressing malignancies [37]–[40]. In addition it has been proposed to have immunosuppressive effects, including the inhibition of lymphocyte proliferation [37]. iNOS is expressed by endothelial cells [41] as well as by arterial wall smooth muscle cells [42]. The finding of increased NM-MRP 8/14 and iNOS in the islets of ES patients is therefore consistent with the previously observed anti-tumorigenic profile in the islets of these patients. Our findings should ideally be validated in another series.

In summary, we have shown that cells other than macrophages express proteins associated with M1 and M2 macrophages. The NM expression of these proteins and their relationship to clinical outcome is again critically dependent on their microlocalisation within the tumour. The cellular expression is also very important as shown previously for TNF [19] and for VEGF in this study. Thus patients who are islet macrophage-VEGF^high^ NM-VEGF^low^ have ES, whereas those who are islet macrophage-VEGF^low^, NM-VEGF^high^ have PS. This has important implications for understanding the biology of these molecules and the delivery of novel biological therapeutics in NSCLC.
