# Correction: The Tissue Microlocalisation and Cellular Expression of CD163, VEGF, HLA-DR, iNOS, and MRP 8/14 Is Correlated to Clinical Outcome in NSCLC

**DOI:** 10.1371/annotation/2088e0ac-4cdb-442d-9259-062f582e8097

**Published:** 2011-11-28

**Authors:** Chandra M. Ohri, Aarti Shikotra, Ruth H. Green, David A. Waller, Peter Bradding

Figure 1A in this article was previously reported in the publication below, cited as reference 14.

Eur Respir J. 2009 Jan;33(1):118-26.

Macrophages within NSCLC tumour islets are predominantly of a cytotoxic M1 phenotype associated with extended survival.

Ohri CM, Shikotra A, Green RH, Waller DA, Bradding P.

The figure is subject to copyright by the European Respiratory Journal. PLoS ONE requires that all figures and images are published under the Creative Commons Attribution License and as a result, we are

removing Figure 1A from this article.

The authors apologise to the readers and editors for any inconvenience caused.

